# Retroperitoneal Laparoscopic Management of Paraganglioma: A Single Institute Experience

**DOI:** 10.1371/journal.pone.0149433

**Published:** 2016-02-17

**Authors:** Weifeng Xu, Hanzhong Li, Zhigang Ji, Weigang Yan, Yushi Zhang, Xuebin Zhang, Qian Li

**Affiliations:** 1 Department of Urology, Peking Union Medical College Hospital, Chinese Academy of Medical Sciences, Beijing, 100730, China; 2 Department of Radiology, Massachusetts General Hospital, Harvard Medical School, Boston, Massachusetts, United States of America; University of Michigan, UNITED STATES

## Abstract

**Objectives:**

To explore the feasibility and safety of retroperitoneal laparoscopic resection of paraganglioma (RLPG) in a large study population.

**Methods:**

In a six-year period, 49 patients with primary retroperitoneal paragangliomas (PG) underwent retroperitoneal laparoscopic surgery in a single center. Medical records were reviewed, and collected the following data, which were clinical characteristics, perioperative data (operative time, estimated blood loss, intraoperative hemodynamic changes, intraoperative and postoperative complications, and open conversions), and follow-up data (recurrence or distant metastases).

**Results:**

All PGs were removed with negative tumor margin confirmed by postoperative histopathology. The operative time of RLPG was 101.59±31.12 minutes, and the estimated blood loss was 169.78±176.70ml. Intraoperative hypertensive and hypotensive episodes occurred in 25 cases and 27 cases, respectively. Two open conversions occurred. Two intraoperative complications occurred but were successfully managed endoscopically. Postoperative complications were minor and unremarkable. No local recurrence or distant metastasis were observed during the follow-up period.

**Conclusions:**

Our experience indicates the feasibility and safety of resection of PGs in a relatively large study population.

## Introduction

Paraganglioma (PG), also known as extra-adrenal pheochromocytoma, is a chromaffin cell tumor located at various sites along the sympathetic/parasympathetic chain, ranging in incidence from 0.005% to 0.1% in the general population[1]. More than 85% of the PGs occur below the diaphragm and most of them are functional, with symptoms and signs of catecholamine overproduction, similar to pheochromocytoma (PCC) except for the variation in the anatomic location[2, 3].

The superiority of laparoscopic surgery for PCC compared with open surgery has been demonstrated, such as less postoperative pain, rapid convalescence, short hospital stay, and improved cosmetic results[4–8]. Open exploration and resection is the standard surgical management of PG, however, laparoscopic resection of PG is considered challenging because of the altered anatomic location, dense peritumoral adhesions, high vascularity envelope, and proximity to major blood vessels. Due to the rarity of the entity, only a few studies of laparoscopic surgery of PG have been reported, most of which were single-case reports or limited case series[3, 9–15]. To the best of our knowledge, most of these studies reported successful removal of PGs, but simultaneously found longer operative time and higher incidence of complications compared with PCC[13–15].

Compared with the transperitoneal laparoscopic approach, which is commonly used to resect retroperitoneal tumors[13–15],the retroperitoneal laparoscopic approach has a few advantages, including shorter operative time, fewer disturbances to abdominal organs, and more rapid convalescence[8, 16–18]. A few studies explored the feasibility of retroperitoneal laparoscopic resection of PG (RLPG)[11, 12, 19], and most of them reported successful outcomes. However, the limited sample size of these studies may introduce unpredictable bias to influence the results’ generalization.

In this retrospective study, we presented our experience of RLPG in a relatively large study population, and explored its feasibility, safety, and surgical outcomes.

## Patients and Methods

### Patients

This retrospective study was approved by the Institutional Review Board of Peking Union Medical College Hospital.

We retrospectively searched our database of medical records from June 2008 to June 2014 and retrieved the subjects at Peking Union Medical College Hospital. Patients satisfying the following criteria were included: (1) primary single retroperitoneal paraganglioma established by postoperative pathology, and (2) laparoscopic surgeries performed via retroperitoneal approach by the same surgical team. Subjects with the following criteria were excluded: (1) confirmed preoperative metastasis, (2) insufficient perioperative data, (3) recurrent PGs, or (4) any previous surgical intervention close to the lesion. Preoperatively, all the subjects to undergo RLPG were consent to take open surgery as an alternative when necessary.

**Fig 1:**
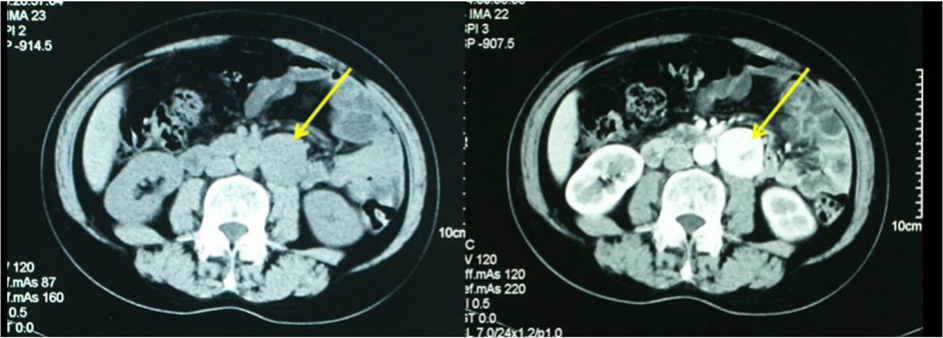
Computed tomography (CT) revealed retroperitoneal paraganglioma(yellow arrow) in the left side.

Over a six-year study period, 49 qualified patients with PG were enrolled in the study. All patients’ catecholamine levels in 24-hour urine samples were measured preoperatively. All the retroperitoneal masses were preoperatively assessed by computed tomography (CT) (Fig 1) and metaiodobenzylguanidine (MIBG) scintigraphy for surgical planning.

The preoperative diagnosis of PGs were made by monitoring 24-hour urine catecholamine level and MIBG scintigraphy. Of all the 49 cases, 41 had elevated catecholamine level or positive result of MIBG scintigraphyand were diagnosed as PGs before operation. The other 8 cases with negative result of catecholamine and MIBG scintigraphy were diagnosed as suspected PGs preoperatively according to clinical symptoms (hypertension/sweating/palpitation) and tumor characteristics in CT scan (location and obvious enhancement). The diagsosisof PGs were confirmed for all 49 cases by postoperative pathology.

### Preoperative preparation of the patients

All the enrolled subjects received α-adrenergic blockade (phenoxybenzamine) at least two weeks before surgery, starting with a dose of 10 mg per day and gradually increasing to 30–90 mg per day. The surgical prerequisites included stable blood pressure (below 140/90 mm Hg) and heart rate (<90 beats per minute) for at least one week. β-adrenergic blockade was instituted if tachycardia occurred following α-adrenergic blockade. The last phenoxybenzamine was administered on the morning of the operation day.

### Surgical equipment

Commonly, we choose 30°endoscopy rather than 0°ones, because the former one has a wider observation range. Ultrasound knifeis the most commonly used energy platform during surgery. Hem-o-lok clip could be used to clamp large vessels, while Hook-electric could help fine dissection. Moreover, titanium clip and laparoscopic needle holder should be prepared as backup.

### Surgical technique

**Fig 2:**
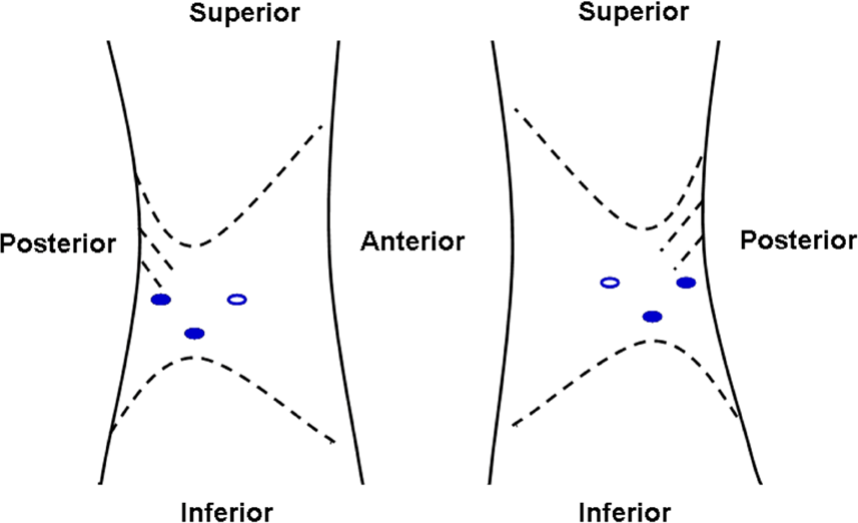
The retroperitoneal laparoscopic approach for PG.

Under general anesthesia, patients were placed in the lateral decubitus position with the lesion side up. The table was flexed with the kidney bridge elevated. The retroperitoneoscopic technique was performed as described by the same team previously[20]. A 1.5-cm incision was made 2 cm above the iliac crest along the midaxillary line. A muscle-splitting-by-finger technique was used. Two trocars were inserted into the retroperitoneum with assistance from the finger. The 10-mm trocar and 5-mm trocar were placed slightly under the costal margin along the posterior and anterior axillary line, respectively (Fig 2). A laparoscope was inserted into the retroperitoneam through the incision above the iliac crest. Carbon dioxide was insufflated to 1.60–1.87 kPa (12–14 mm Hg) and was used throughout the operation.

**Fig 3:**
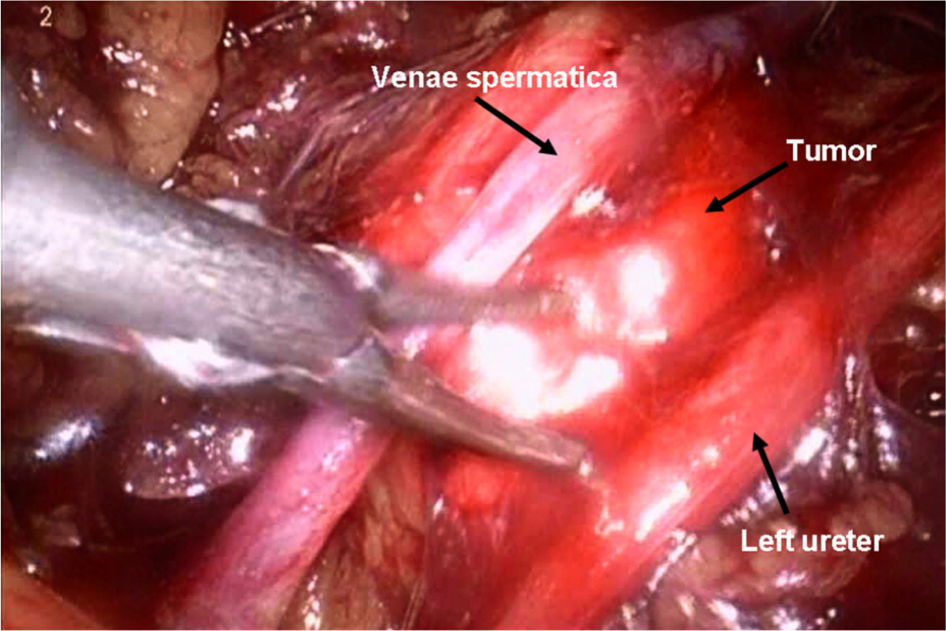
Retroperitoneal laparoscopic resection of PG.

In the pneumoperitoneum, the retroperitoneal fat should be pushed down and an incision made in the Gerota’s fascia anterior to the psoas muscle. The kidney was fully mobilized until the tumor was clearly visualized considering their tight anatomic relationship. A fourth trocar which was placed anterior superiorly to the first incision was sometimes necessary to retract the kidney. The PG has no consistent blood supply patterns. Major vessels and small branches should be carefully identified and separated and sealed by ultrasonic scalpel or electrocoagulation, sometimes clamped by hem-o-lok clips. The tumors are then completely dissected from the surrounding tissue and the large vessels nearby(Fig 3). The incision was enlarged based on the tumor size, and the tumor was removed through a retrieval bag.

### Perioperative and follow-up data collection

The hospital charts and operative notes of enrolled subjects were reviewed retrospectively, along with the baseline characteristics and the surgical outcomes. A “hypertensive episode” was defined as an increased systolilc blood pressure (SBP) by 30% above the baseline level or SBP of 200mmHg or higher. A ‘‘hypotensive episode” was defined as SBP that decreased below 80mmHg[14]. Postoperative surgical complications within 1 month were recorded and evaluated according to Clavien-Dindo’sclassification[21].

Postoperatively, all patients were followed up 1 month, and subsequently, monitored by computed tomography and 24-hour urine catecholamine levels every 3–6 months. Any local recurrence or distant metastasis was recorded during the follow-up period.

### Statistical analysis

All statistical analyses were conducted with SPSS® software, version 17.0 (SPSS, Inc., Chicago, IL, USA). Continuous variables, such as age, BMI, tumor size, preoperative preparation duration, catecholamine levels in 24-hour urine, operative time, estimated blood loss, and postoperative hospital stay were expressed as mean ± SD.

## Results

### 1. Baseline characteristics of enrolled subjects

As described in Table 1, the subjects’ baseline profile and tumor-associated data were summarized, including sex, age, BMI, tumor size, tumor location, ASA grade, preoperative 24-hour urine catecholamine level, and preoperative preparation duration.

**Table 1:** Clinical Demographics and Tumor Characteristics.

	RLPG^a^ (n = 49)
Age	37.10±14.33
Male/Female	21/28
Body mass index (kg/m^2^)	24.80±1.41
Tumor diameter (cm)^b^	4.53±1.18
Location (left/right)	27/22
ASA^c^ grade (Grades I-II/III)	39/10
Preoperative preparation duration (α-blocker: days):	24.82±5.33
Preoperative urinary hormone	
Norepinephrine (μg/24h)	157.43±165.01
Epinephrine (μg/24h)	3.38±1.89
Dopamine (μg/24h)	176.18±55.79
MIBG^d^ (positive/negative)	26/23

^a^retroperitoneal laparoscopic resection for retroperitoneal paragangliomas

^b^Based on preoperative CT/magnetic resonance imaging (MRI)

^c^American Society of Anesthesiologists grade

^d^Metaiodobenzylguanidine scintigraphy

### 2. The symptoms of the PGs

Of all the preoperational symptoms of PGs (Table 2), the hypertension was the most common (77%), following with sweating (53%), palpitation (45%), etc.

**Table 2:** The overall preoperative symptoms of PGs.

Symptoms	n (%)	95%CI (%)
Hypertension	38 (77%)	63.4–88.2
Persistent hypertension	31 (63%)	-
Paroxysmal hypertension	7 (14%)	-
Sweating	26 (53%)	38.3–67.4
Palpitation	22 (45%)	30.7–59.8
Headache	15 (31%)	18.3–45.4
Anxiety	5 (10%)	3.4–22.2

*one-sided 95%CI

### 3. The sizes and locations of the PGs

**Fig 4:**
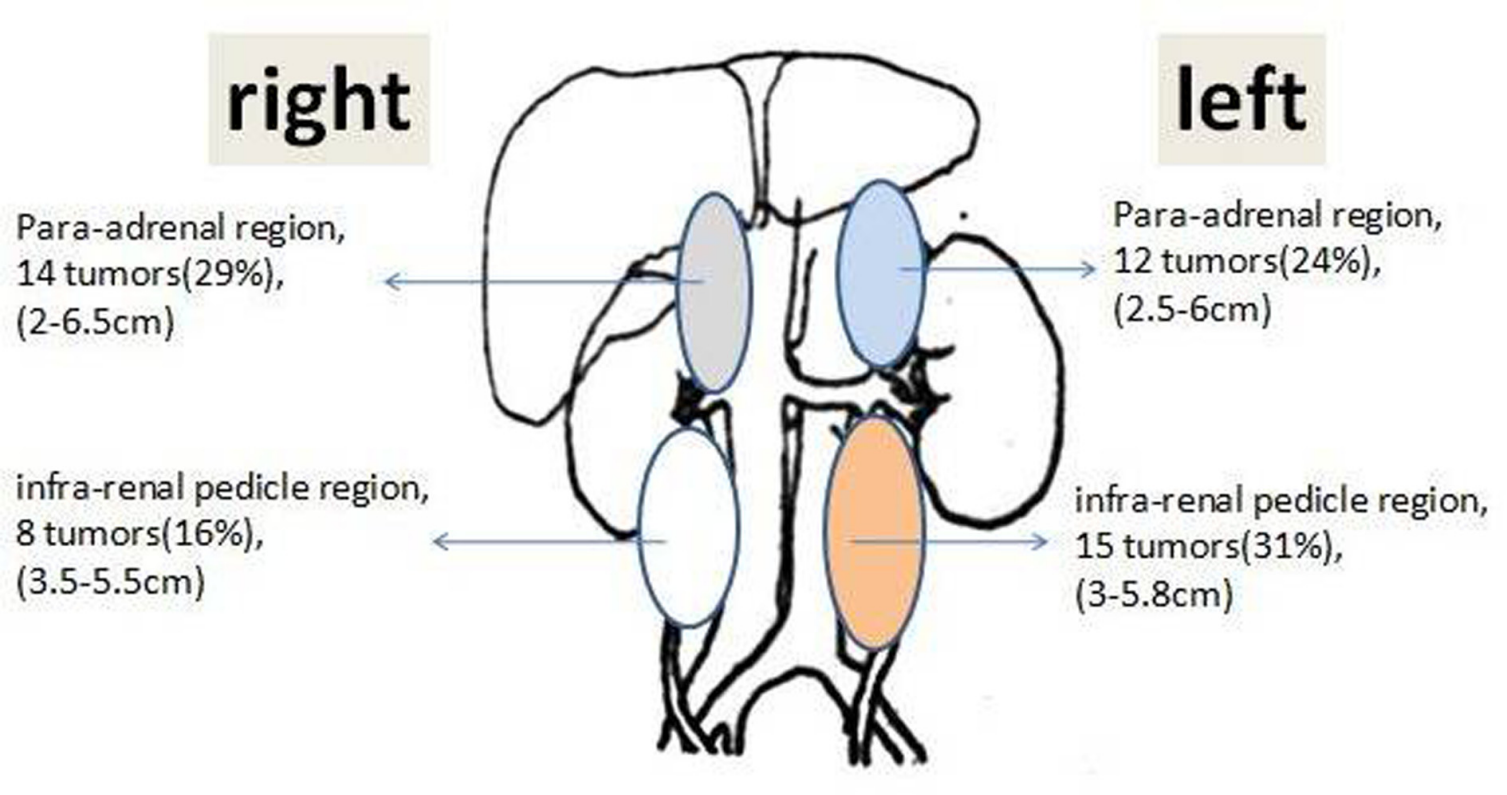
The anatomic diagram of the tumors. The lesions were located in the four regions of retroperitoneal space with similar size ranges and frequencies (29%, 24%, 16%, and 31%).

The anatomic diagram (Fig 4) showed sizes and numbers of lesion in different regions of retroperitoneal space.

### 4. Perioperative Data

The perioperative data were summarized in Table 3.

**Table 3:** Perioperative Data of RLPG Group.

	RLPG^a^ (N = 49)
Open conversion (cases)	2
Operative time (min)*	101.59±31.12
Estimated blood loss*	169.78±176.70
Blood transfusion (cases)	3
Intraoperative hemodynamic changes	
Incidence of hypertensive episode	25/49
Incidence of hypotensive episode	27/49
Intraoperative complications (cases)	2
Postoperative hospital stay (days) *	5.81±0.77
Postoperative complications (cases) **	5
Grade I	3
Grade II	2

^a^retroperitoneal laparoscopic resection of retroperitoneal paragangliomas

*N = 47, cases with open conversion were excluded

**Clavien-Dindo classification

In this study, two open conversions occurred because of dense adhesion between the tumors and inferior vena cava (IVC), and both open procedures were subsequently performed successfully without intraoperative blood transfusions or other complications. All the PGs were resected with negative tumor margins, confirmed by postoperative histopathology.

The operative time and estimated blood loss were analyzed in the 47 cases who underwent successful RLPG, which were 101.59±31.12 minutes and169.78±176.70 ml respectively. Intraoperative blood transfusion occurred in 3 procedures because of relatively large blood loss (600–1100ml), and these three PGs were all resected successfully via retroperitoneal approach without conversions to open operations. The maximum diameters of the three PGs were 5.0cm, 5.8cm, and 6.5cm, which all showed dense adhesion to surrounding tissuesor great vessels. The postoperative hospital stay was 5.81±0.77 days.

Two intraoperative complications occurred in this study. In one case, an accessory renal artery (1-mm in diameter) crossing the tumor was transected by Hem-o-lok clips, leading toa small pale area of the renal lower pole, but no serious consequences occurred. In another case, a3-mm sidewall rupture of the renal vein occurred, but was closed successfully by a titanium clip. Five postoperative complications were observed in this study, including 3 Clavien Grade I (1 wound infection and 2 lymphorrhagia) and 2 Clavien Grade II (1 deep venous thrombosis of lower extremity and 1 pneumonia). All of the complications were treated with conservative therapy, and finally recovered.

The follow-up interval ranged from 6 to 55 months (median 31 month), during which, no local recurrence or distant metastasis occurred.

## Discussion

Although the safety and efficacy of laparoscopic adrenalectomy for PCC have been well documented in recent years, studies of laparoscopicexcision of PGs are a total of 8 retrospective studies with limited sample sizes and 23 case reports between 1998 and 2013[11]. Of the 84 patients enrolled in these studies, 3 underwent robot-assisted operation, 68 underwent transperitoneal laparoscopic resection, and 13 underwent retroperitoneal laparoscopic resection. The laparoscopic method has been demonstrated feasible for PGs, but still challenging due to longer operative time and higher incidence of postoperative complications, especially for RLPG[3, 11–14]. In addition to the fewer publication numbers, the reported data fluctuated greatly due to the limited sample size. By evaluating the success rate of RLPG, intraoperative and postoperative complications, intraoperative hemodynamic changes, and follow-up outcomes, our data suggested that retroperitoneal laparoscopic surgery was feasible and safe for resection of PGs based on the most populous RLPG enrollment (n = 49).

In this study, only two conversions to open surgery occurred due to the dense adhesion of PGs to IVC other than surgical technical issues. Careful evaluation based on findings of CT, MIBG, classic clinical symptoms, and elevation of 24-hour urinary catecholamine, provides no unanimous conclusion of the nature of tumor characteristics. Therefore, an alternate open surgery of the mass adhering to important organs or large vessels was necessary to avoid major complications[22]. Two intraoperative complications in this study were attributed to dense adhesion of tumor to the renal vessels, but fortunately, both were successfully managed endoscopically. Compare with open surgery, laparoscopic surgery is more accurate in dissection, especially when the tumor is closely related to the renal pedicle, laparoscopic surgery is more competitive for kidney pedicle protection, and thereby avoid forced kidney removal. Base on our experience, when venous injury occured during laprascopic surgery, we could simply increasingpneumoperitoneum pressure by 2 KPa (15mmHg) to stop further bleeding, and suture/clamp the breakage under a relatively clear operation field.

Five minor postoperative complications were observed (wound infection, lymphorrhagia, deep venous thrombosis of lower extremity, and pneumonia), which were also occasionally encountered in general endoscopic procedure, but not solely associated with the procedural approach.[14, 23]

Altered intraoperative hemodynamics is another important factor to evaluate the safety of the PGs resection, as 25% to 60% of the PGs are functional with symptoms and signs of catecholamine overproduction[2, 3]. In this study, no unmanageable hypertensive or hypotensive episodes occurred during the RLPG, which demonstrated that the retroperitoneal surgery did not induce the disastrous overproduction of catecholamines in PG. Our experience showed that fully preoperative pharmacological preparation was the key to success, and the strategy similar to PCC was also appropriate for PG. Additionally, the follow-up data showed no recurrence or metastasis within at least 6 months after operation in this series, which indicated that RLPG was possibly acceptable in terms of prognosis.

In our opinion, the key to a successful RLPG lay in the surgical team, primarily. Due to the diversity of the lesion sites and the complicated relationship with great vessels, laparoscopic resection of PG was a high-risk procedure, and suggested to be performed by senior surgeons with adequate laparoscopic experiences. Our surgical team had experience of more than 250 laparoscopic resections of PCCs or PGs, and was sufficiently familiar with the adrenal tumor anatomy and well trained on laparoscopic operation, which was critical for the RLPG success.

PGs are characterized with enriched blood supply and variation of the tumor vessels. So, isolation of the tumor should be performed carefully to avoid errhysisand tumor-feeding vessels should be well ligated. Meanwhile, the manipulationon the tumor should be gentle to avoid the rupture of the capsule, which will consequently lead to bleeding of tumor body, even conversion to open surgery. Based on our experiences, no special surgical equipment was used in RLPG compared with other laparoscopic procedures.

The transperitoneal approach was the mainstay of the laparoscopic procedures because of the broad working space and maximal tumor exposure. However, in our department, the retroperitoneal method has been routinely used for PCC since 2003 due to advantages of safety, cost-effectiveness, and diminished risk of abdominal injury. Based on our experience, we preferred the retroperitoneoscopicapproachfor patients with PG, which offered direct access to tumors without mobilizing the abdominal organs, thus minimizing the risk of injury to abdominal viscera and reducing operative time. Although the smaller retroperitoneal working space and less anatomic landmarks are challenging, our data showed that retroperitoneal approach was feasible to expose most of PGs less than 8 cm in diameter and visualize the adjacent major vessels, which was consistent with previous reports.[20]

Operative time and estimated blood loss are both important indicators to assess the efficacy of a procedure. In several studies, which enrolled 5–9 PGs with smaller size (3.3–4.8 cm)[3, 13, 14], the operative time of transperitoneal laparoscopic resection ranged from 189.8 to 290.4 min, and the estimated blood loss was 108–1036.3ml. By contrast, our study demonstrated relatively shorter operative time (101.6±31.1 min) and lower estimated blood loss (169.8±176.7ml) of RLPG, possibly due to the direct access to tumor via the retroperitoneal approach and surgical expertise of the urological team. One study involving 10 RLPG cases demonstrated similar operative time (mean 97.8 min) as ours (mean 101.5 min)[20], but our data are definitely more representative because of the larger sample size. Additionally, in our study, intraoperative blood transfusion was required in 3 cases due to large amount of blood loss (600–1100ml). These tumors were not only relatively large (5cm, 5.8cm, and 6.5cm), but also densely adhered to surrounding tissue and great vessels, which raised the complexity of tumor dissection.

The first limitation of this study was related to its retrospective nature, which will introduce uncontrolled recall bias and confounders, inevitably affecting the results. Second, the follow-up interval was relatively short, thus further investigation is warranted to track the surgical results for the long run. Finally, as a challenging surgical technique, the indications for RLPG in this study were strictly controlled by the surgeons, which will introduce a selection bias.

Our six-year experience of RLPG indicated its feasibility and safety when performed by skilled urologists, which provided direct tumor access, less intraperitoneal interference, precise dissection, and minimal invasiveness. The results warranted substantial motivation to further investigate RLPG as a promising technique to manage PGs.
